# The ROP vesicle release factor is required in adult Drosophila glia for normal circadian behavior

**DOI:** 10.3389/fncel.2015.00256

**Published:** 2015-07-03

**Authors:** Fanny S. Ng, F. Rob Jackson

**Affiliations:** Department of Neuroscience, Sackler School of Biomedical Sciences, Tufts University School of MedicineBoston, MA, USA

**Keywords:** glia, Drosophila, SNARE, syntaxin, ROP/Munc18, circadian

## Abstract

We previously showed that endocytosis and/or vesicle recycling mechanisms are essential in adult Drosophila glial cells for the neuronal control of circadian locomotor activity. In this study, our goal was to identify specific glial vesicle trafficking, recycling, or release factors that are required for rhythmic behavior. From a glia-specific, RNAi-based genetic screen, we identified eight glial factors that are required for normally robust circadian rhythms in either a light-dark cycle or in constant dark conditions. In particular, we show that conditional knockdown of the ROP vesicle release factor in adult glial cells results in arrhythmic behavior. Immunostaining for ROP reveals reduced protein in glial cell processes and an accumulation of the Par Domain Protein 1ε (PDP1ε) clock output protein in the small lateral clock neurons. These results suggest that glia modulate rhythmic circadian behavior by secretion of factors that act on clock neurons to regulate a clock output factor.

## Introduction

During the past decade, it has become apparent that glial cells of vertebrates and invertebrates are active modulators of neuronal development, synaptogenesis, and excitability, in addition to having metabolic support roles (reviewed in Allen, 2014; Jones and Bouvier, 2014; Freeman, 2015). Factors released from mammalian astrocytes, for example, including Thrombospondins, SPARC, Glypicans 4 and 6, C1q and TNFα have active roles in synaptogenesis, synapse elimination, or glia-neuron signaling (Christopherson et al., 2005; Stevens et al., 2007; Jones et al., 2011; Santello et al., 2011; Allen et al., 2012; Santello and Volterra, 2012). Similarly, Drosophila glial cells secrete proteins such as Myoglianin, Wingless (WG), and Maverick (MAV) that regulate neuronal morphogenesis or synapse formation/function (Awasaki et al., 2011; Fuentes-Medel et al., 2012; Kerr et al., 2014). Proteomic studies have suggested that hundreds of other proteins are secreted from mammalian glia (Lafon-Cazal et al., 2003; Delcourt et al., 2005; Dowell et al., 2009; Keene et al., 2009; Moore et al., 2009), and certain of these proteins are known to regulate synapse formation or neuronal activities (reviewed by Jones and Bouvier, 2014). In addition to secreted proteins, previous studies have suggested that glia release their own transmitters (“gliotransmitters”) including D-serine, glutamate, and ATP, via SNARE-dependent mechanisms, to modulate neuronal excitability and plasticity (reviewed by Araque et al., 2014; Covelo and Araque, 2015; Haydon and Nedergaard, 2015; Zorec et al., 2015).

Vesicle release mechanisms and the relevant proteins are highly conserved between species (reviewed by Jahn, 2000; Lloyd et al., 2000; Hong and Lev, 2014). Although proteins involved in exocytosis have been best characterized in neurons, some may have similar functions in glial astrocytes (Gucek et al., 2012). For example, Syntaxin (syx) SNARE family members and a Syx-binding partner (ROP/Munc18) are known to regulate neuronal vesicle release in Drosophila and mammals, (Garcia et al., 1994; Harrison et al., 1994; Halachmi et al., 1995; Fujita et al., 1996; Wu et al., 1998, 1999, 2001), but Syntaxins, Munc18 and other SNAREs are also expressed in “active zones” of glial astrocytes, and their functions in glia may be related to vesicle release (Paco et al., 2009; Schubert et al., 2011; Tao-Cheng et al., 2015). A number of studies indicate that glial secretion mechanisms and glia-neuron interactions are also relevant for the regulation of rhythmic behaviors such as sleep and circadian locomotor activity (reviewed in Jackson et al., 2015).

Studies in mammals and Drosophila indicate that circadian mechanisms can influence or be influenced by glial astrocyte functions. For example, mammalian astrocytes rhythmically release ATP, cytokines and growth factors, although it is unclear whether vesicle release mechanisms are required for these rhythms (reviewed in Jackson et al., 2015). It is also known that there are diurnal changes in the astrocytic coverage of Vasointestinal Polypeptide (VIP) neurons of the suprachiasmatic nuclei that are associated with changes in synaptic innervation of these neurons (SCN; Serviere and Lavialle, 1996; Girardet et al., 2010). Our previous studies suggest that glial proteins and normal glial functions are required for regulation of circadian rhythms in locomotor activity (Suh and Jackson, 2007; Ng et al., 2011). Conditional, adult perturbation of glial endocytosis and vesicle recycling mechanisms, for example, led to arrhythmic circadian behavior and dysregulation of neuronal Pigment Dispensing Factor (PDF), an important circadian neurotransmitter (Ng et al., 2011). These results indicate an important physiological role for glia-neuron signaling in circadian behavior.

As vesicle recycling and release mechanisms are highly conserved, we have employed Drosophila as a genetic model to study the involvement of such mechanisms in glia-neuron communication and circadian behavior. In the current study, we investigated roles for specific glial vesicle trafficking and release factors in viability and behavior. We examined 37 different genes known to encode vesicle trafficking or release factors and to be expressed in glial cells of mammals or flies (Lovatt et al., 2007; Cahoy et al., 2008; Huang et al., 2015). We performed a targeted RNAi-based screen to determine whether such factors are required for viability, locomotor activity, or circadian behavior. We found that ten genes are essential in glial cells for fly development; 10 others are required for normal locomotor activity or rhythmic behavior. These genes encode proteins important for exocytosis, endocytosis, and vesicle trafficking between the ER and Golgi. For example, we show that adult, glial-specific knockdown of ROP/Munc18 (vesicle release factor) or Syntaxin 5 (ER to Golgi trafficking) expression results in arrhythmic behavior. These results suggest that both glial vesicle trafficking and release are essential for maintaining circadian rhythmic behavior. Our studies also demonstrate that perturbation of glial vesicle release results in altered neuronal PDP1ε abundance, indicating a role for glia-neuron signaling in clock output.

## Materials and methods

### Fly strains and culture conditions

All Drosophila cultures were maintained on standard media in a light:dark cycle (LD) consisting of 12 h of light and 12 h of dark (LD 12:12). Experimental flies were generated by crossing virgin females of the Gal4 line to males of UAS-IR lines. Control flies were obtained from a cross of Gal4 or UAS-IR flies to flies of the genetic background strains of the UAS-IR or Gal4 lines. For constitutive pan-glial knockdown experiments, the repoGal4 transgenic line was used to induce glial RNAi expression (UAS-IR); crosses were reared at 25°C. The tubG80^ts^, repoGal4 or tubG80^ts^, alrmGal4 transgenic lines were used to conditionally activate glial RNAi expression under high temperature (30°C); crosses were reared and flies entrained at 23°C to prevent the transcriptional activation of UAS-IR during development. We have shown that tubG80^ts^, repoGal4>UAS-DsRed and tubG80^ts^, alrmGal4>UAS-DsRed do not have detectable DsRed signal at 23°C. In contrast, there is a high level of DsRed signal at 30°C (data not shown). UAS-IR transgenic lines for *kermit* (*GIPC*), *amph, scamp, syx1*, comatose (*dNSF-1*), *unc-13, Snap25, exo 70, syx 16, syx 17, syx7, syx 5, synapsin, syx 18, cDase, cg*12811, *Bet 1, Bet 5, Ykt 6, cg*1968, *cg*31232, *syx 6, syx 8, gammaSnap, vamp 7, syt 4, aplip 1, wkd, AP-2sigma, orange, cg*10703, *Rop, sec 23, sec 5, sec 6, sec 13, WDR 79* were obtained from Harvard TRiP, via the Bloomington stock center, or the Vienna Drosophila Resource Center (VDRC).

### Collection of locomotor activity and data analysis

Flies less than 1 week old were placed in Trikinetics Drosophila Activity Monitors housed in a temperature-controlled incubator. In each experiment, locomotor activity was monitored for 20 days, with 4–5 days of LD 12:12 entrainment followed by 10–15 days in free-running conditions (constant dark, DD) at a specific temperature (see Results). Behavioral data (beam breaks) were collected in 30-min bins and then analyzed using the MATLAB-based signal processing toolbox (Levine et al., 2002). Percent rhythmicity for populations was determined via the rhythmicity index (RI, a measurement of robustness) and the correlogram (a statistical measurement of rhythmicity), as previously described (Ng et al., 2011).

### Immunohistochemistry

Four to six individual males of experimental and control strains were selected according to their behavioral profile during the first 6 days of DD at a specific temperature and time of day (see Figure legends). Flies were immobilized on ice and brains hand dissected in ice-cold PBS in the dark under red light. After both experimental and control strains were dissected, brains were fixed with ice-cold 4% paraformaldehyde (PFA) on ice for 25 min. Three ice-cold PBS washes were used to remove any residual PFA. Then a 10 min incubation of PBS with 0.05% Triton-X100 (PBST) on ice was used to facilitate antibody penetration of tissue. Brains were incubated with 5% normal goat serum (in PBST) for 3 h on ice to block non-specific antibody binding. Brains were incubated with primary antibody for 2 days at 4°C, followed by 1 day of secondary antibody incubation at 4°C after PBST washes. All samples were mounted with Vectashield (Vector laboratories) to preserve the secondary fluor. To detect ROP, BRUCHPILOT, and NAZGUL (NAZ) proteins, the following antibodies were used: rabbit anti-NAZ (1:800–1:1000, a gift from B. Altenheim), mouse anti-ROP (4F8, 1:800, Developmental Studies Hybridoma Bank, DSHB), mouse anti-BRUCHPILOT (nc 82, 1:100, DSHB). To detect specific circadian clock proteins, the following antibodies were used: rabbit anti-PER (1:7500, a gift of R. Stanewsky); guinea pig anti-PDP1 (1:30,000, a gift from Dr. P. Hardin); and mouse anti-PDF (1:100, DSHB). The PER antibody was preabsorbed against per^01^ embryos to eliminate any non-specific signal. Relevant secondary antibodies were employed at 1:800. These included Alexa Fluor 488, goat anti-rabbit; Alexa Fluor 488, goat anti-mouse; Cy3, goat anti-guinea pig; Cy3, goat anti-rabbit; Cy3, goat anti-mouse; and Alexa Fluor 647, goat anti-rabbit. All Alexa Fluor secondary antibodies were purchased from Invitrogen; the Cy3, goat anti-rabbit secondary was obtained from Jackson ImmunoResearch Laboratories. Confocal images were acquired with a Leica TCS SP2 AOBS microscope for co-immunostaining of ROP and NAZ. All other image acquisition was performed using a Leica SPE microscope. For all experiments, control, and experimental brains were examined at the same time with similar acquisition parameters.

Fuji ImageJ 1.47v was used to generate projected images from optical sections of small ventral lateral neurons (sLNv) or the whole brain. Two or three independent experiments were performed for each study, and images were quantified without knowing the genotype. Before performing any intensity quantification, background subtraction- the standard Rolling Ball plug-in with radius as 50 pixels—was used. Intensity was measured by overlaying the Region of interest (ROI) with the background subtracted images. ROIs were defined based on three different applications: (1) Intensity in sLNv cell body: the PDF signal was used as marker for outlining by hand. (2) Intensity along dorsal projections of sLNv: first any signals within the 60 μm distance from the center of cell bodies were eliminated on the max projection images, then intensities of these processed images were transformed by using the Otsu threshold method, and finally the ROIs were outlined from these final images. (3) Intensity within the NAZ processes: all optical section images with NAZ signals were transformed by the Otsu threshold method; the Analyze Particles plug-in was used to outline areas on these processed images with sizes between two to five micron^2^ (non-circular). The images shown in figures were further processed with Adobe Photoshop.

### Statistical analysis

To assess statistical significance of circadian behavior parameters and of immunostaining signal intensities, we used a Kruskal–Wallis test (non-parametric ANOVA) with Dunn's Multiple Comparison test if the data did not pass a normality test (using the method of Kolmogorov and Smirnov). One-Way ANOVA with Tukey–Kramer Multiple Comparisons test was used if the data passed a normality test (Instat, GraphPad). A two-tailed *t*-test was used to assess statistical significance for PDF, PDP, and PER immunostaining signals in the sLNv neurons.

## Results

### Glial vesicle trafficking and/or secretion mechanisms are important for viability and rhythmic behavior

We previously showed that glia of the adult *Drosophila* nervous system, in particular astrocytes, are essential for regulation of circadian behavior (Ng et al., 2011): conditional, glia-specific manipulations of several different cellular processes, including vesicle release/recycling, were associated with arrhythmic circadian locomotor activity. In the present study, we have extended these findings by asking whether specific vesicle trafficking mechanisms are required for adult rhythmic behavior. Based on the results of several different studies that investigated gene expression profiling of glial cells in mammals or *Drosophila* (Table S4) (Freeman et al., 2003; Altenhein et al., 2006; Lovatt et al., 2007; Cahoy et al., 2008; Kim et al., 2010; Huang et al., 2015), we selected 37 genes which are known to be involved in vesicle intracellular trafficking, secretion, endocytosis, or recycling. To study the roles of the encoded factors in regulating circadian behavior, we performed glial cell-specific RNA interference (RNAi)-based knockdowns for the relevant genes, using the fly Gal4/UAS binary expression system (reviewed by St Johnston, 2002), and monitored circadian locomotor activity rhythms (see Materials and Methods). In our initial screen, we utilized the pan-glial, repoGal4 driver to selectively regulate expression of various UAS-RNAi transgenes in glial cells of the brain. As most of the selected genes are known to be essential for development, we monitored both viability and behavior in flies constitutively expressing RNAi transgenes throughout development.

**Figure 1:**
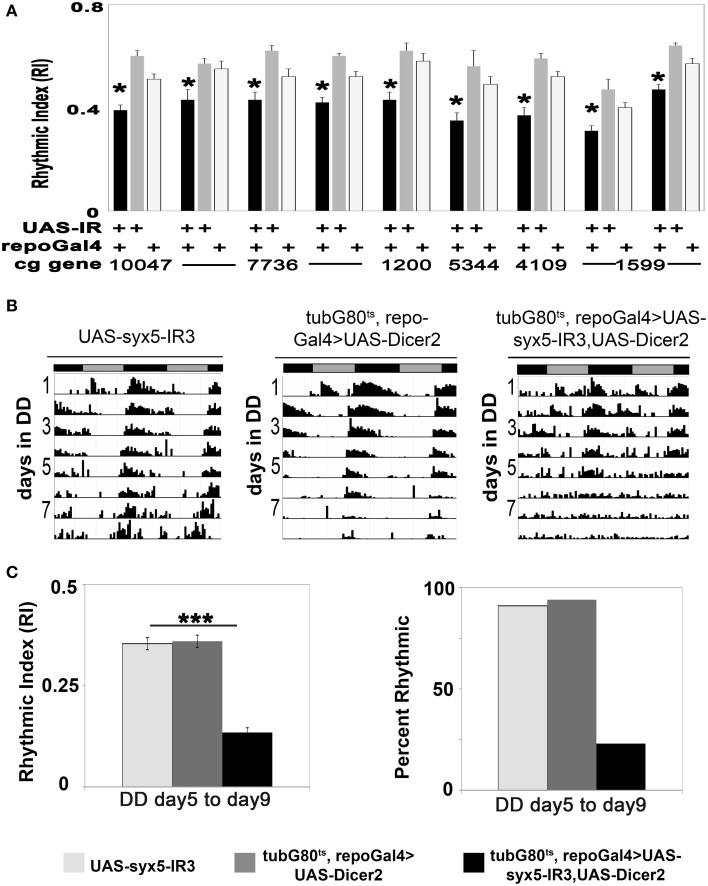
**Glial vesicular protein functions are important for neuronal control of circadian locomotor activity. (A)** Histogram summarizing the reduction in RI value for experimental strains with constitutive glial knockdown under DD conditions. The experimental strain (repoGal4>UAS-IR) is represented by the black column, the control strains (UAS-IR/+), and (repoGal4/+) are represented by gray and light gray columns respectively. **(B)** Representative actograms for control flies or individuals with the conditional expression of UAS-syx5-IR. Activity was collected in DD at 30.5°C. UAS-syx5-IR3 represents Bloomington stock #29397. **(C)** Histograms summarizing the RI value and percent of rhythmic flies in experimental and control populations. *p < 0.05; ***p < 0.001 (One-Way ANOVA) compared with both control strains. Error bars represent SEM. Data was collected from two independent experiments, and all data are included in Tables S1, S2.

Among the 89 independent RNAi transgenes (UAS-IR) that we examined, the expression of 49 resulted in altered development or behavior (Table 1, Tables S1–S3). For example, expression of a number of sec and syntaxin (syx) UAS-IR transgenes, including Rop, sec5, 6, and 23, Bet1, 5 or Ykt6 or syx1, 5 and 8, using repoGal4, resulted in lethality prior to eclosion (Table 1). Thus, the glial functions of these genes are crucial for development. Pan-glial expression of other UAS-IR transgenes were not lethal but caused small reductions in locomotor activity or the robustness of rhythmicity (RI) in LD or constant darkness (DD) conditions; these included Synaptotagmin 4 (syt4), syx6 and 8, APP-like protein interacting protein 1 (aplip1), whacked (wkd), vesicle-associated membrane protein 7 (vamp7), γ Soluble NSF attachment protein 1(gammaSnap1), and Adaptor Protein complex 2-σ subunit (AP-2sigma) (Table 1, and Table S1; Figure 1A; Morel et al., unpublished results). Although the defects observed with individual gene deficits were not as severe as those seen with complete disruption of endocytosis/exocytosis (Ng et al., 2011), they confirm that normal glial vesicle trafficking, either during development or in adults, is required for rhythmic locomotor activity.

**Table 1:** **Behavioral and viability phenotypes of UAS-IR-expressing strains**.

**Phenotypic deficits under constitutive pan-glial knockdown (*repo*Gal4)**	**Gene**
Reduction in activity level during LD	*cg*7736*(syx6*), *cg*4109(*syx8*)
Reduction in RI value during LD	*cg*7736(*syx6*), *cg*3988 (*gammaSnap*), *cg*1599*(vamp7)*, *cg*1958
Reduction in RI value during DD	*cg*10047(*syt4*), *cg*7736(*syx6*), *cg*1200(*aplil1*), *cg*5344(*wkd*), *cg*4109(*syx8*), *cg*1599(*vamp7*), *cg*6056(AP-2sigma)
Lethality	*cg*15811(*Rop*), *cg*1250(*sec23*), *cg*8843(*sec5*), *cg*5341(*sec6*), *cg*4109(*syx8*), *cg*4214(*syx5*), *cg*1359*(Bet5)*, *cg*14084*(Bet1)*, *cg*1515*(Ykt6)*, *cg*31136 *(syx1)*
**PHENOTYPIC DEFICITS UNDER CONDITIONAL ADULT PAN-GLIAL KNOCKDOWN (*tub*G80^ts^, *repo*Gal4 or *tub*G80^ts^, *repo*Gal4 > UAS Dicer2)**	
Reduction in RI value and in percent rhythmicity during high temperature DD	*cg*15811(*Rop*)
Lethality, reduction in RI value and in percent rhythmicity during high temperature DD	*cg*4214(*syx5*)

*IR transgenes were expressed throughout development (top) or conditionally in adulthood (bottom)*.

### Sec1 and syntaxin homologs are required in adult glial cells for normal circadian locomotor activity rhythms

Studies in mammals and Drosophila have shown that similar mechanisms regulate neurotransmitter vesicle trafficking, fusion, and release (reviewed by Lloyd et al., 2000; Yoshihara et al., 2001; Hong and Lev, 2014). To determine whether vesicle trafficking/release factors are required in adult glial cells, we conditionally expressed UAS-IR transgenes targeting *Rop*, *sec5/6/23*, *Bet1/5, Ykt6, syx1/5/6/8*, *gammaSnap*, *aplip1*, *wkd*, *vamp7*, *syt4*, *cg1968*, and *AP-2sigma*. These studies were performed using the so-called TARGET system (McGuire et al., 2004), which takes advantage of a temperature-sensitive form of a Gal4 inhibitor (Gal80^ts^) to turn on transgene expression in the adult brain after development. At temperatures below 23°C, Gal80^ts^ inhibits Gal4 whereas at elevated temperatures (≥ 29°C) it is inactivated and Gal4 expression ensues. For our experiments, we generated fly strains (tub-G80^ts^, repoGal4>UAS-IR) in which G80^ts^ is ubiquitously expressed. To avoid the activation of RNAi during development, we raised tub-G80^ts^, repoGal4>UAS-IR, and control populations at 23°C and then monitored locomotor activity of adult flies at 30.5°C.

The SNARE protein Syx5 functions in vesicle trafficking between ER and Golgi (Bard et al., 2006; Kondylis et al., 2011) and thus deficits are expected to alter vesicle secretion and/or recycling. Using three independent UAS-syx-IR transgenes (3859, 108928, and 29397), we monitored behavioral rhythms in tub-G80^ts^, repoGal4>UAS-syx-IR, and control flies at low and high temperatures. Pan-glial expression of each of the three UAS-syx-IR transgenes resulted in effects on adult viability or circadian rhythms. With one exception, adult tub-G80^ts^, repoGal4>UAS-syx-IR flies died within 2 days of high temperature treatment, whereas control flies had normal viability. Flies of the exceptional strain, tub-G80^ts^, repoGal4>UAS-syx5-IR3-29397, survived at high temperature for at least 11 days, indicating a less potent effect of the IR transgene. However, with addition of UAS-Dicer2 in the background of this strain (tubG80^ts^, repoGal4>UAS-syx5-IR3-29397, UAS-Dicer2), only 23% of the population remained rhythmic, with an average RI value of 0.13 (Figures 1B,C; Table S2). This contrasts with control populations (tubG80^ts^, repoGal4>UAS-Dicer2, and UAS-syx5-IR3-29397) which were rhythmic with average RI values of 0.36 and 0.35, respectively at high temperature (Figures 1B,C, Table S2; *p* < 0.001 compared to experimental strains). Importantly, both experimental and control flies entrained and were normally rhythmic at low temperatures (Table S2). These results suggest that SYX5 function is essential in glial cells of the adult brain for normal rhythmic behavior.

**Figure 2:**
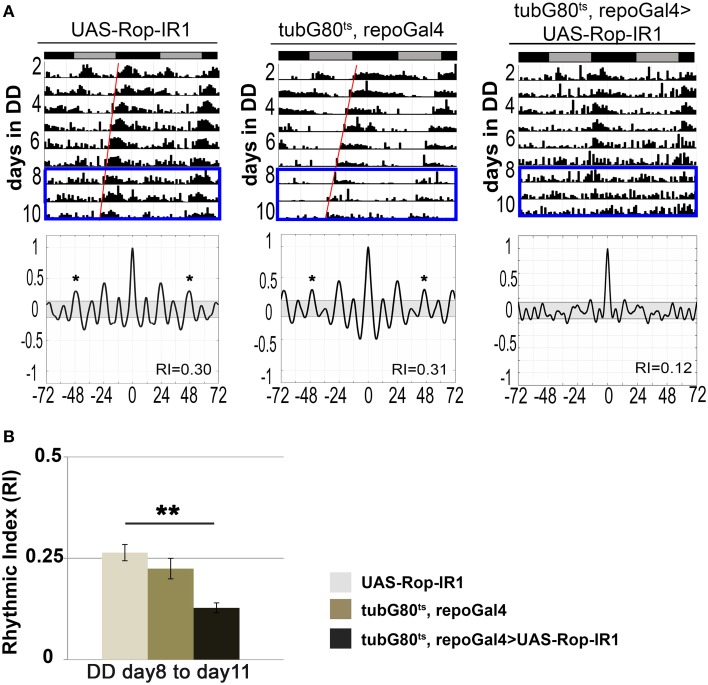
**ROP protein function is required for circadian locomotor activity. (A)** Actograms showing the first 11 days of activity from 14-day DD experiments at 30.5°C (top). Each actogram is a representative record for each genotype. Bottom panels show the corresponding correlograms for the truncated (8–11 day) records highlighted with a blue rectangle in the actograms (top). The RI value for each actogram is shown in the corresponding correlogram. *p < 0.05 (Correlogram analysis). **(B)** Histogram summarizing changes in RI value for conditional adult glial knockdown of *Rop* compared with control strains. Data was collected from at least three independent experiments. **p < 0.01 (One-Way ANOVA), and SEM is indicated by error bars. All data are included in Table S3.

Studies of the conserved sec1 homolog ROP/Munc18 have revealed functions in neuronal vesicle fusion and release (Harrison et al., 1994; Schulze et al., 1994 and reviewed by Halachmi and Lev, 1996). For example, adult visual responses and excitatory junction potentials (EJPs) at larval neuromuscular junctions are reduced in several Rop mutant flies (Harrison et al., 1994; Schulze et al., 1994; Wu et al., 1998). In contrast to results with constitutive expression (see above), conditional adult expression of any one of three UAS-Rop-IR transgenes (28929, 106242, and 19696) did not affect viability, and flies survived until the end of activity experiments. Among the three, UAS-Rop-IR1 (28929) produced circadian arrhythmicity. Less than half (48%) of tubG80^ts^, repoGal4>UAS-Rop-IR1 flies exhibited rhythmic locomotor activity by the end of the experiment (day 8–11 of DD), and the average RI was 0.13 (*p* < 0.01, compared with control strains). Under the same conditions, ≥70% of the control strains exhibited rhythmic behavior with average RIs ≥0.22 (Figures 2A,B; Table S3). In addition, both experimental and control flies displayed relatively normal rhythmicity at low temperatures (Table S3). Presumably the difference among UAS-Rop-IR transgenes is due to the effectiveness of ROP knockdown. When UAS-Rop-IR1 was expressed in astrocyte-like glia, arrhythmic behavior was observed in a small percentage of this experimental strain (tubG80^ts^, alrmGal4>UAS-Rop-IR1). However, the average RI was not significantly different from control populations (Table S3). We attribute the lack of effect to the observation that alrmGal4 is a weaker driver than repoGal4. We have directly documented a weaker expression of alrmGal4 in a ventral group of glial cells using a UAS-CD8::GFP transgene (data not shown). Rop-IR-28929 is not predicted to have off-target effects (based on the use of dsCheck software—http://dscheck.rnai.jp/), and we show below that ROP protein is decreased with expression of the IR transgene. These observations suggest a physiological role for ROP in glia of the adult brain.

### ROP is expressed in glia of synaptic-rich regions of the adult brain

**Figure 3:**
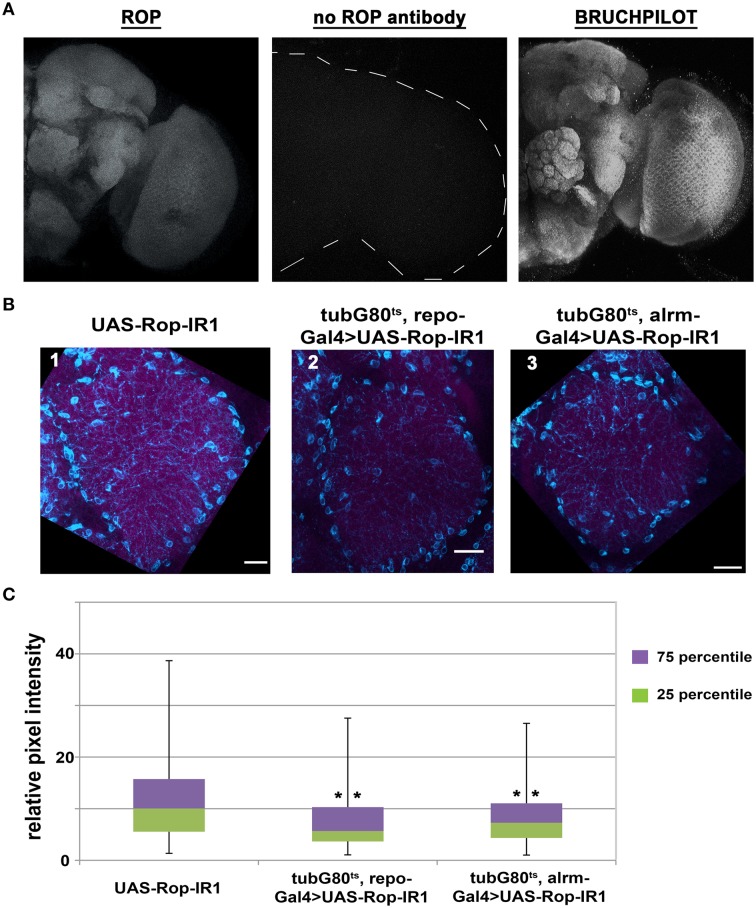
**ROP can be detected in synapse-rich areas and its level is reduced with glial expression of UAS-Rop-IR. (A)** Maximum z-projections of confocal images of whole adult brains acquired with a 40X objective and 1.5 μm as the optical Z step. Left panel: a representative image of ROP immunostaining. Middle panel: secondary goat anti-mouse antibody alone, control. Right panel: a representative image for BRUCHPILOT immunostaining. **(B)** Maximum z-projection confocal images (~9 μm) of co-staining with anti-ROP (magenta) and anti-NAZ (cyan) in Ventrolateral Protocerebrum (VLP) from fly brains dissected on the 7th day of DD at 30.5°C. Scale bar represents 10 μm in length. Images were acquired using a 60X objective with 2.5 digital zoom and 1 μm as the optical Z step. Three genotypes are shown: UAS-Rop-IR1 (panel 1, IR1); tubG80^ts^, repoGal4>UAS-Rop-IR1 (panel 2, TRIR1); tubG80^ts^, alrmGal4>UAS-Rop-IR1 (panel 3, TAIR1). Images shown here are all enhanced by Photoshop with the following adjustments in threshold and hue filters; threshold minimum and maximum for all channels are set to 19 and 227 respectively, filter for hue is set to be +13. **(C)** Box plots quantifying signal intensities for ROP within the NAZ+ glial processes. Twenty-four different brain images (eight for each genotype) were collected from three independent experiments. 2566, 1232, and 1490 ROIs are examined in IR1, TRIR1, and TAIR1, respectively. The error bars represent maximum and minimum intensity for a given genotype. The 75th percentile of the distribution is shown as purple and the 25th percentile as green. **p < 0.001 (non-parametric One-Way ANOVA) when comparing TRIR1 or TAIR1 with IR1 and between TRIR1 and TAIR1.

From previous studies (Harrison et al., 1994; Schulze et al., 1994), it is known that ROP is present in muscle, secretory tissues, and neuropil of the third-instar larval central nervous system. ROP can also be detected by immunoblot analysis in adult head tissues, but its spatial expression pattern in adult brains is unknown. Therefore, we used a ROP-specific antibody (4F8) and immunofluorescence techniques to examine the expression of ROP in adult brains. This work revealed that ROP is broadly expressed in the adult brain, and that its expression pattern is very similar to BRUCHPILOT (nc82) antibody (Figure 3A), which is present at synaptic endings (Wagh et al., 2006). Similarly, ROP appears to be present in synaptic-rich regions of the adult brain. Notably, in flies with pan-glial expression of UAS-Rop-IR1, there was an apparent decrease in ROP signal (Figure 3B; compare 2 to 1). Expression of the Rop-IR in astrocyte-like glia (using alrmGal4) also resulted in a small decrease in the ROP signal (Figure 3B; compare 3 to 1); in contrast, overexpression of ROP resulted in an increased ROP signal in synaptic-rich regions of the brain (Figure S1). These brain regions also contain neuronal processes and thus the change in ROP within glial cells could not easily be quantified by examination of the entire neuropil.

To further characterize the ROP signal, and to be able to verify knockdown and overexpression of the protein, we looked for markers that labeled glial processes. An antibody against NAZGUL (NAZ), a glial marker of unknown function, detects the protein in a subpopulation of interface glial cells which are probably neuropil glia (Figure S2; von Hilchen et al., 2010 and B. Altenheim, pers. comm.). Unlike ROP, which is strongly detected in neuropilar processes of wild-type flies, NAZ is localized in glial cells bodies surrounding the neuropil and in glial processes that infiltrate the neuropil (Figure 3B and Figure S1). The absence of ROP in NAZ-containing cell bodies is readily apparent when outlines are drawn around the cell bodies (Figure S2B). However, as already indicated, there is an obvious increase in ROP staining intensity, relative to controls, with repoGal4-driven expression a transgene encoding wild-type ROP, and in addition signal was now detected in cell bodies of the NAZ glial population (Figure S1). We conclude that Rop is present in NAZ-containing glia.

NAZ and alrm cells belong to the class of interface glia, and they both have processes that extend into the neuropil (Figure 3B and Doherty et al., 2009), To simultaneously image NAZ and alrm-positive glial processes, we immunostained brains of alrmGal4>UAS-mCD8::GFP adults with the anti-NAZ antibody. Such flies express a membrane-tethered form of GFP in alrm-positive glia, and this permits visualization of glial processes. We found that a majority of NAZ+ cell and its processes were also labeled with GFP (Figure S3), indicating that NAZ and alrm are expressed in the same glial cell population.

### ROP is present in processes of NAZ cells and is decreased in amount with pan-glial UAS-Rop-IR expression

Flies with conditional, pan-glial expression of a UAS-Rop-IR transgene exhibit altered circadian behavior (Figure 2). To verify that ROP is reduced in amount in these flies, we examined ROP immunoreactivity in NAZ glial processes under the same conditions. As already indicated (Figure S1), NAZ antibody labeled both the cell body and glial processes. To discriminate these, we used Fuji Image J software to delineate the NAZ cell processes, and outlines of these processes were used as regions of interest to examine ROP intensity. This approach permitted quantification of changes in ROP immunoreactivity in the processes of a subpopulation of glial cells. As shown in Figure 3C, less anti-ROP signal was detected in glial processes of Rop-IR-expressing flies compared with the control. Similarly, there was a small but significant reduction in ROP with conditional, alrmGal4-driven expression of Rop-IR (Figure 3C). These results indicate that reduced ROP function in glia is responsible for the arrhythmic behavior of Rop-IR flies.

### Glial ROP knockdown results in a constant high level of par domain protein (PDP1) in sLNv neurons

**Figure 4:**
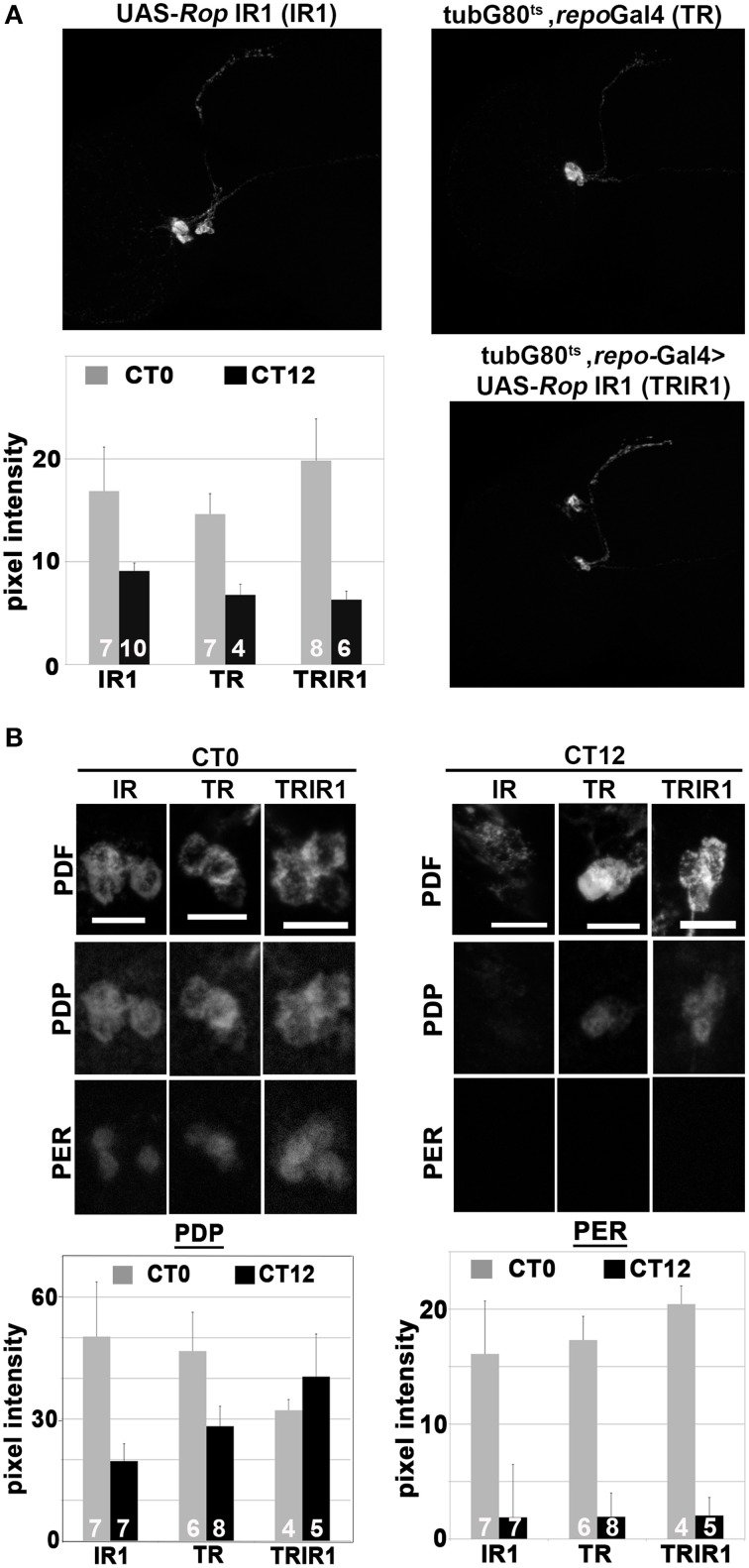
**Glial expression of UAS-Rop-IR results in constant high levels of PDP1 in sLNv neurons but does not affect PER**. Three genotypes are shown: UAS-Rop-IR1 (IR1); tubG80^ts^, repoGal4 (TR); tubG80^ts^, repoGal4>UAS-Rop-IR1 (TRIR1). To examine PDF, PER, and PDP1 cycling expression, fly brains were collected at CT0 (subjective dawn) and CT12 (subjective dusk) on the 7th day of DD at 30.5°C. **(A)** Representative maximum z-projection confocal images of PDF immunostaining of adult brains at CT0. Histogram summarizes the PDF signal intensity along the dorsal projections (starting at 60 μm away from the cell body). SEM is indicated by error bars. PDF intensity is significantly different (*p* < 0.05, two-tailed *t*-test) between CT0 and CT12 for each genotype. However, there are no statistically significant differences in PDF intensities among genotypes at CT0 or CT12. **(B)** Top panels: Representative confocal images of PDF (top row), PDP1 (middle row), and PERIOD (PER, bottom row) in sLNv neurons at CT0 and CT12. Bottom panels: Histograms quantifying PDP1 and PER intensities in the sLNv neurons at CT0 and CT12 in the different genotypes. Left histograms show that PDP1 pixel intensity at CT0 is different from CT12 in IR1 and TR (*p* < 0.05, two-tailed *t*-test). There is no significant difference in PDP1 signal between CT0 and CT12 in TRIR1. Right histograms show that PER pixel intensity is significantly different between CT0 and CT12 in all genotypes.

Drosophila PDF, a circadian neuropeptide released from the sLNv clock cell processes, is essential for rhythmic locomotor activity (reviewed by Shafer and Yao, 2014). In a previous study, we showed that PDF immunoreactivity is abolished, in a reversible manner, by conditional perturbation of glial vesicle release and recycling (Ng et al., 2011). This result can be interpreted as an effect on PDF release. Since ROP is required for vesicle docking and release, reduced ROP function may result in a similar effect on PDF. Therefore, we examined PDF intensity in the axonal projections of the sLNv processes during DD day 7 at CT0 (time of peak PDF) and CT12 (trough of PDF intensity). However, we found no significant differences in PDF levels between the experimental and control strains at either time point. Furthermore, PDF intensity in the sLNv projections showed robust cycling in both types of flies (Figure 4A). Thus, it seems unlikely that the arrhythmicity observed in flies with reduced glial ROP is caused by abnormal PDF release from the sLNv neurons.

To try to understand how glial ROP deficits cause arrhythmicity, we examined cycling of PERIOD (PER), a core component of the circadian oscillator, and PDP1ε, a cycling transcription factor implicated in clock function and output (Benito et al., 2007; Zheng et al., 2009). Examination of PER in the sLNv neurons during DD day 7 revealed a robust rhythm of immunoreactivity, with an approximate eight-fold difference between the peak and trough of the clock protein even with conditional glial knockdown of ROP (Figure 4B). In contrast, levels of PDP1 were similar at CT0 and CT12 in the experimental strain, whereas a two-fold difference was observed in the control strains. However, the constant high level of PDP1 seems not to affect the timing of nuclear entry (Figure 4B). These results suggest that reduced glial ROP function causes abnormal circadian behavior by altering PDP1 neuronal expression.

## Discussion

Studies in mammals and *Drosophila* have shown that glia, in particular astrocytes, can modulate neuronal activity or behavior in the adult brain (reviewed in Zwarts et al., 2014; Haydon and Nedergaard, 2015). In a previous study, we showed that glial vesicle recycling mechanisms are critical for maintenance of circadian behavior (Ng et al., 2011): conditional glial-specific perturbations of vesicle recycling in adult Drosophila, using a temperature-sensitive, dominant negative dynamin, resulted in arrhythmic behavior. The current study was aimed at gaining additional insights about the glial vesicle trafficking mechanisms which regulate the circadian neuronal circuit and rhythmic behavior. We performed a targeted glial-specific RNAi-based screen of genes encoding factors that control vesicle secretion, intracellular vesicle trafficking, endocytosis, or vesicle recycling. Pan-glial expression of UAS-RNAi (UAS-IR) transgenes targeting 37 different genes identified 17 that are required for viability or normal circadian behavior (Table 1). Targeting of *sec6*, *sec23*, *rop*, *Ykt6*, *Bet1*, *Bet5*, *syx1*, or *syx5* resulted in developmental lethality, not surprising given the known role of glia in *Drosophila* nervous system development. In contrast, constitutive glial expression of UAS-IR transgenes for *gammaSnap*, *wkd*, *vamp7*, *AP-2sigma*, and *syx6* throughout development did not cause lethality but resulted in effects on the robustness of adult circadian behavior (Table 1 and Table S1; Morel et al., unpublished results). Phenotypes were observed with multiple, different UAS-IRs for each of these genes, making it less likely that they result from off-target effects.

In addition to having roles in Drosophila embryonic neural development (Crews, 2010), it has been shown that glial functions are required for normal neuromuscular junction formation, neuronal excitability and a number of adult behaviors (reviewed in Zwarts et al., 2014). Thus, we asked if glial expression of any of the 17 identified genes was required in the adult brain for normal viability or circadian behavior. Conditional adult glial expression of two different UAS-syx5-IR transgenes resulted in lethality within 2 days, indicative of a physiological requirement for the gene product, but excluding circadian behavioral analysis. Conditional glial expression of a third UAS-syx5-IR and one UAS-Rop-IR were associated with arrhythmic behavior. Thus, both ROP and SYX5, which have known roles in vesicle trafficking or secretion, are required in glia of the adult nervous system for normal circadian behavior (Figures 1, 2; Tables S2, S3).

SYX5 is necessary for cargo transport between the ER and Golgi, and deficits for the protein result in fusion of the two intracellular compartments (Bard et al., 2006; Kondylis et al., 2011). Thus, effects on adult viability are not unexpected. We assume the behavioral phenotype observed with conditional expression of one UAS-syx5-IR is a consequence of a less severe (“hypomorphic”) reduction in activity of the SNARE, which may result in effects on the trafficking and/or secretion of an essential glial factor.

As mentioned above, our RNAi-based screen showed that constitutive and conditional glial expression of UAS-Rop-IRs caused developmental lethality and arrhythmic circadian behavior, respectively. These findings demonstrate that glial ROP is required physiologically in the adult brain in addition to being critical for development. We show that ROP is localized to adult synaptic-rich regions and along the processes of NAZ-positive interface glial cells. Since ROP is known to be expressed in neuronal synaptic endings (Harrison et al., 1994; Schulze et al., 1994), it is of interest that the protein is also present in glial processes. Several previous studies have suggested that Rop and the mouse ortholog Munc18 are expressed in glial cells (Paco et al., 2009), but the current study is the first to reveal the spatial expression of ROP protein in glia of the adult brain. In neurons of *Drosophila* and mammals, ROP/MUNC18 regulates synaptic vesicle fusion and release mechanisms through interaction with other vesicle associated proteins (Weimer and Richmond, 2005; reviewed by Jahn, 2000; Hong and Lev, 2014). Moreover, studies of null mutants have shown that fly ROP is also required for general secretion mechanisms in non-excitable cells (Harrison et al., 1994; Halachmi et al., 1995; reviewed by Halachmi and Lev, 1996). Hence, we postulate that ROP may serve a similar function in glial cells, perhaps regulating secretion of so-called “gliotransmitters” (reviewed in Araque et al., 2014) or other factors (e.g., secreted proteins). *Drosophila* glial cells secrete many factors, including WG and MAV that have been implicated in synapse maturation and function (Fuentes-Medel et al., 2012; Kerr et al., 2014).

Rhythmic locomotor activity is one behavioral output of the circadian neuronal circuit. From a multitude of studies, it is known that individual clock neurons of both flies and mammals contain a self-sustaining molecular oscillator. In flies, the circadian clock relies on activities of several proteins including CLK, CYC, PER, TIM, VRI, PDP1ε, and CWO (Hardin, 2011). The population of clock neurons comprising the circadian circuit is synchronized by release of several peptide neurotransmitters from the sLNv and other neurons (Helfrich-Forster et al., 2000, 2013; Johard et al., 2009; Hermann et al., 2012; Hermann-Luibl et al., 2014). In particular, loss of the Pigment Dispersing Factor (PDF) peptide—a critical circadian neurotransmitter—from LNv neurons leads to arrhythmic behavior (Renn et al., 1999; Helfrich-Forster et al., 2000; Park et al., 2000). Previously, we showed that conditional and reversible perturbations of glial vesicle recycling altered PDF immunoreactivity in the projections of the sLNv clock neurons. Surprisingly, conditional glial deficits for ROP, which affect behavior, do not alter PDF intensity or cycling (Figure 4A). Normal rhythmic release of the neuropeptide implies that neuronal clock mechanisms are functional, and indeed we observed strong cycling of PER protein in sLNv neurons of ROP knockdown flies. Thus, there is no evidence for effects on the core clock mechanism (the CLK-CYC/PER-TIM loops). However, we observed a constant high level of PDP1ε in the sLNv neurons of UAS-Rop-IR-expressing flies, in contrast to controls which exhibited normal cycling of the protein. Of note, it has previously been shown that constant levels of the PDP1ε transcription factor—induced by overexpression in clock neurons—leads to behavioral arrhythmicity without altering the central clock mechanism or PDF (Benito et al., 2007). This result suggests that PDP1ε can alter clock output with no effects on the circadian molecular oscillator or PDF.

The high level of PDP1ε observed in UAS-Rop-IR flies suggests that the protein may not be degraded properly. Degradation of PDP1ε is promoted by its own phosphorylation state, and doubletime (DBT or CK1ε) is known to phosphorylate the protein both *in vitro* and *in vivo* in a clock-independent manner (Choi et al., 2009). Perhaps a slow accumulation of non-degraded PDP1ε in clock neurons—induced by conditional glial knockdown of ROP and altered glia-neuron signaling—leads to the gradual deterioration of rhythmic behavior observed in our studies. This, of course, begs the question of how alterations of PDP1ε in clock neurons affect rhythmic behavior in a PDF-independent manner. While such a mechanism must involve release of another factor from clock neurons (perhaps encoded by a PDP1ε target gene), that factor currently remains unidentified.

## Conflict of interest statement

The authors declare that the research was conducted in the absence of any commercial or financial relationships that could be construed as a potential conflict of interest.
