# Supplementary material for: The ROP vesicle release factor is required in adult Drosophila glia for normal circadian behavior
Source: Front Cell Neurosci. 2015 Jul 3;9:256. doi: 10.3389/fncel.2015.00256 (PMC4490253; doi:10.3389/fncel.2015.00256)
Supplement: Supplementary file 1 [file DataSheet1.PDF]

Supplementary Material

The ROP vesicle release factor is required in adult Drosophila glia for normal circadian behavior

Fanny S. Ng and F. Rob Jackson*

Department of Neuroscience, Tufts University School of Medicine, Sackler School of Biomedical Sciences,136 Harrison Avenue, Boston MA 02111

*** Correspondence:** Corresponding author at the address above
Email – rob.jackson@tufts.edu; Tel. 617-636-6752

1. Supplementary Data

There are four tables and three figures included in this supplementary data sheet.

Table S1.	Locomotor activity parameters for control flies and those expressing UAS-IR transgenes.
Table S2.	Locomotor activity parameters for flies expressing a UAS-syx5 IR transgene and controls.
Table S3.	Locomotor activity parameters for flies expressing a UAS-Rop IR transgene and controls.
Table S4.	Genes examined in the pan-glial RNAi screen.
Figure S1.	ROP immunoreactivity is detectable in NAZ cell bodies with overexpression of a wild-type UAS-Rop transgene.
Figure S2.	ROP is preferentially expressed at a high level in synapse-rich regions containing NAZ processes.
Figure S3.	NAZ and alrm are expressed in the same subpopulation of adult glial cells.

2.1 Supplementary Tables

Supplementary Table 1. Locomotor activity parameters for control flies and those expressing UAS-IR transgenes.

A. Activity level and entrainment of strains with pan-glial expression of RNAi transgenes.

Gene	Predicted off target	Genotype	LD (at least 3 days)		
			N	mean activity level ± sem	percent Entrainment
*cg*7736(*syx6*)	*cg*7413	repoGal4>104795	32	17.98 ± 0.87 *	78%
		104795	32	22.95 ± 1.09	100%
		repoGal4	30	22.68 ± 1.50	100%
*cg*4109(*syx8*)	*cg*32592	repoGal4>42561	32	21.34 ± 0.76*	88%
		42561	32	29.33 ± 1.56	100%
		repoGal4	32	28.02 ± 1.20	100%

Conditions: LD 12:12, 25°C.

B. Average RI values for repoGal4>RNAi strains at 25°C in LD 12:12.

Gene	Predicted off target	Genotype	LD (at least 3 days)		
			N	RI ± sem	percent Entrainment
*cg*7736 (*syx6*)	*cg*7413	repoGal4>104795	32	0.24 ± 0.02 *	78%
		104795	32	0.34 ± 0.02	100%
		repoGal4	30	0.34 ± 0.02	100%
	n/a	repoGal4>28505	27	0.31 ± 0.02 *	70%
		28505	30	0.41± 0.01	100%
		repoGal4	28	0.38 ± 0.02	100%
*cg*3988 *(gammaSnap)*	n/a	repoGal4>26346	16	0.44 ± 0.03 *	88%
		26346	16	0.55 ± 0.02	100%
		repoGal4	43	0.52 ± 0.02	98%
*cg*1968	n/a	repoGal4>20767	26	0.28 ± 0.03 *	100%
		20767	29	0.39 ± 0.03	100%
		repoGal4	32	0.42 ± 0.03	100%
*cg*1599(*vamp7*)	n/a	repoGal4>13317	47	0.44 ± 0.01*	98%
		13317	46	0.53 ± 0.01	100%
		repoGal4	43	0.52 ± 0.02	98%

C. Average RI values or repoGal4>RNAi strains at 25°C in DD.

Gene	Predicted off target	Genotype	DD (at least 10days)		
			N	RI ± sem	percent Rhythmic
*cg*10047 (*syt4*)	*cg*33512	repoGal4> 33317	32	0.39± 0.02 *	100%
		33317	29	0.60 ± 0.02	100%
		repoGal4	31	0.51 ± 0.03	100%
*cg*7736 (*syx6*)	n/a	repoGal4>28505	19	0.43 ± 0.04 *	84%
		28505	30	0.57 ± 0.02	100%
		repoGal4	27	0.55 ± 0.03	100%
	*cg*7413	repoGal4>1501	32	0.42 ± 0.02 *	100%
		1501	31	0.60 ± 0.01	100%
		repoGal4	30	0.52 ± 0.02	100%
*cg*1200 (*aplip 1*)	*cg*5583	repoGal4>109501	16	0.43 ± 0.03 *	100%
		109501	16	0.62 ± 0.03	100%
		repoGal4	15	0.58 ± 0.03	100%
*cg*5344 (*wkd*)	n/a	repoGal4>22081-v	26	0.35 ± 0.03 *	80%
		22081-v	30	0.56 ± 0.06	97%
		repoGal4(v)	29	0.49± 0.03	100%
*cg*4109 (*syx8*)	*cg*32592	repoGal4>42561	28	0.37 ± 0.03 *	90%
		42561	30	0.59 ± 0.02	100%
		repoGal4	31	0.52 ± 0.02	100%
*cg*1599 (*vamp7*)	n/a	repoGal4>13316-v	17	0.31 ± 0.02 *	82%
		13316-v	18	0.47 ± 0.04	94%
		repoGal4	21	0.40 ± 0.02	100%
	n/a	repoGal4>13317	28	0.47 ± 0.02 *	82%
		13317	39	0.64 ± 0.01	100%
		repoGal4	26	0.57± 0.02	100%

Data were collected from at least two independent experiments. The specific RNAi strains used in the experiments are included in the genotype columns. Asterisks (*) indicate experimental strains with significantly different parameters ($p<0.05$, one way ANOVA) when compared with both the Gal4 and UAS-IR control strains. (**A**) Activity level and entrainment of strains with pan-glial expression of RNAi transgenes. (**B**) Average RI values for repoGal4>RNAi strains at 25°C in LD 12:12. (**C**) Average RI values for repoGal4>RNAi strains at 25°C in DD.

Supplementary Table 2. Locomotor activity parameters for flies expressing a UAS-syx5 IR transgene and controls.

A. Average activity level, RI values and percent entrainment in LD 12:12 at 23°C.

Genotype	N	mean activity level ± SEM	RI ± SEM	percent Entrainment
UAS-syx5-IR3	64	23.47 ± 0.76	0.36 ± 0.01	100%
tubG80^{ts}, repoGal4 > UAS-Dicer2	64	20.56 ± 0.61	0.34 ± 0.01	100%
tubG80^{ts}, repoGal4> UAS-syx5-IR3,UAS-Dicer2	94	20.97 ± 0.48	0.36 ± 0.01	100%

B. Average activity, circadian period and RI values in DD at 30°C (DD5 to DD9).

Genotype	N	mean activity level ± SEM	period ± SEM	RI ± SEM	percent Rhythmic
UAS-syx5-IR3	32	16.35 ± 0.83	23.46 ± 0.06	0.35 ± 0.02	91%
tubG80^{ts}, repoGal4 > UAS-Dicer2	32	18.61 ± 1.12	23.30 ± 0.10	0.36 ± 0.02	94%
tubG80^{ts}, repoGal4> UAS-syx5-IR3,UAS-Dicer2	61	13.68 ± 0.60*	22.95 ± 0.44	0.13 ± 0.01***	23%

C. Average activity, circadian period and RI values in DD at 23°C (DD day5 to day9).

Genotype	N	mean activity level ± SEM	period ± SEM	RI ± SEM	percent Rhythmic
UAS-syx5-IR3	32	20.30 ± 1.47	23.38 ± 0.08	0.28 ± 0.02	81%
tubG80^{ts}, repoGal4> UAS-Dicer2	26	25.32 ± 1.11	23.78 ± 0.10	0.34 ± 0.02	85%
tubG80^{ts}, repoGal4> UAS-syx5-IR3,UAS-Dicer2	30	24.96 ± 1.79	23.50 ± 0.09	0.31 ± 0.02	80%

Data for repoGal4>UAS-syx5 IR3 (29397) are averages of two independent experiments. (**A**) Average activity levels and RI values in LD 12:12 at 23°C. (**B**) Average activity, circadian period and RI values in DD at 30°C (DD5 to DD9). (**C**) Average activity, circadian period and RI values in DD at 23°C (DD day5 to day9). A one-way ANOVA was used in these experiments and those summarized in Table S3 to determine significant differences between IR-expressing and control flies. *, $p<0.05$; ***, $p<0.001$.

Supplementary Table 3. Locomotor activity parameters for flies expressing a UAS-Rop IR transgene and controls.

A. Average activity levels, RI values and percent entrainment in LD 12:12 at 23°C.

Genotype	N	mean activity level ± SEM	RI ± SEM	percent Entrainment
UAS-Rop-IR1	32	19.72 ± 0.79	0.31 ± 0.01	100%
tubG80^{ts}, repoGal4	48	15.16 ± 0.66	0.35 ± 0.01	98%
tubG80^{ts}, alrmGal4	51	23.66 ± 1.02	0.37 ± 0.02	98%
tubG80^{ts}, repoGal4>UAS-Rop-IR1	76	16.2 ± 0.44	0.26 ± 0.01*	89%
tubG80^{ts}, alrmGal4>UAS-Rop-IR1	60	20 ± 0.85	0.26 ± 0.01	87%

B. Average activity, circadian period and RI values and percent rhythmic in DD at 30°C (DD8 to DD11).

Genotype	N	mean activity level ± SEM	period ± SEM	RI ± SEM	percent Rhythmic
UAS-Rop-IR1	31	14.24 ± 1.11	23.70 ± 0.20	0.26 ± 0.02	84%
tubG80^{ts}, repoGal4	27	10.81 ± 0.85	23.82 ± 0.12	0.22 ± 0.03	70%
tubG80^{ts}, alrmGal4	42	10.86 ± 0.94	23.73 ± 0.21	0.27 ± 0.02	83%
tubG80^{ts}, repoGal4> UAS-Rop-IR1	102	16.45 ± 0.67	23.84 ± 0.18	0.13 ± 0.01**	48%
tubG80^{ts}, alrmGal4> UAS-Rop-IR1	75	14.76 ± 0.63	23.80 ± 0.08	0.22 ± 0.02	65%

C. Average activity, circadian period and RI values and percent rhythmic in DD at 23°C (DD8 to DD11).

Genotype	N	mean activity level± SEM	period ± SEM	RI ± SEM	percent Rhythmic
UAS-Rop-IR1	31	18.36 ± 1.19	23.95 ± 0.06	0.35 ± 0.02	94%
tubG80^{ts}, repoGal4	29	21.21 ± 1.10	23.83 ± 0.03	0.48 ± 0.02	100%
tubG80^{ts}, alrmGal4	42	28.39 ± 1.67	24.10 ± 0.07	0.45 ± 0.02	96%
tubG80^{ts}, repoGal4> UAS-Rop-IR1	60	17.53 ± 0.82	23.77 ± 0.11	0.30 ± 0.01	82%
tubG80^{ts}, alrmGal4> UAS-Rop-IR1	30	12.81 ± 0.75*	24.01± 0.10	0.32 ± 0.02	100%

Data are compiled from at least two independent experiments. (**A**) Average activity levels, RI values and percent entrainment in LD 12:12 at 23°C. (**B**) Average activity, circadian period and RI values and percent

rhythmic in DD at 30°C (DD8 to DD11). (**C**) Average activity, circadian period and RI values and percent rhythmic in DD at 23°C (DD8 to DD11). *, p<0.05; **, p<0.01.

Supplementary Table 4. Genes examined in the pan-glial RNAi screen.

Gene Drosophila (mammal)	References
kermit (GIPC)	2, 4
amph	1, 2, 3
scamp	1, 2
syx1 (stx1)	2
comt	2, 3
unc13	1, 2
snap25	2
exo70	1, 2
syx16 (stx16)	1, 2, 3
syx17(stx17)	2, 3
syx7(stx7)	1, 2
syx5 (stx)	2
synapsin (syn)	2
syx18 (stx18)	2
cDase	1, 3
*cg*12811	3
Bet1	2, 3
Bet5	3
Ykt6	3
*cg*1968	3
*cg*31232	3
syx6 (stx6)	1, 2
syx8 (stx8)	1, 2
gammaSnap	3
vamp7	3
syt4	1, 2
aplip1	3
wkd	3
AP-2sigma	2, 3
orange	3
*cg*10703	3
Rop (munc18)	1, 3
sec23	2, 3
sec5 (exoc2)	2, 3
sec6 (exoc3)	1, 2
sec13	2, 3
WDR79	3

Footnote: 1, Altenhein et al., 2006; 2, Cahoy et al., 2008; Huang et al., 2015; Kim et al., 2010

2.2 Supplementary Figure Legends

Figure S1. ROP immunoreactivity is detectable in NAZ cell bodies with overexpression of a wild-type UAS-Rop transgene. Co-immunostaining of NAZ (green) and ROP (magenta) in 1-um optical sections of adult brain VLP. Top panels show merged images of NAZ and ROP; middle panels are NAZ-only images; bottom panels are ROP-only images. *, indication of cell bodies with both NAZ and ROP signals.

Figure S2. ROP is preferentially expressed at a high level in synapse-rich regions containing NAZ processes. (**A**) ROP (magenta) and NAZ (cyan) merged brain images. Left panel: maximum z-projection of optical sections of whole adult brain. Right panel: zoomed-in version of a single 1-um optical section of the region (white box shown in left panel); "c" indicates locations of NAZ+ cell bodies. (**B**) ROP expression around NAZ cell bodies. Left panel, merged image of ROP and NAZ+ cell outlines generated by the Fuji Image J program (the right-hand image from panel a). ROP is shown in gray and the NAZ+ outline as cyan. An enlargement of the yellow box is presented on the right.

Figure S3. NAZ and alrm are expressed in the same subpopulation of adult glial cells. (**A**) Images showing GFP (green) and NAZ (red) immunostaining in the VLP region from alrmGal4>UAS-mCD8::GFP brains. * indicate areas in which both GFP and NAZ can be detected. Left panel is the merged image of GFP and NAZ; middle panel is GFP alone; right panel is NAZ alone. (**B**) Table showing the number of ROIs (NAZ+ cell) generated by particle analysis in Fuji Image J (column T), and number of ROIs (NAZ+ cell) detected with GFP signals above background (column B). The last column shows the ratio of GFP+ and NAZ+ ROIs with respect to the total number of NAZ+ cells ROIs. Filter parameters for the Analyze Particles plug-in were a cell size within three to five micron^2 which was completely circular. Images shown here are all enhanced by Photoshop with the following adjustments in threshold and hue filters: 1) threshold minimum and maximum for the green channel were set to 3 and 194 respectively, 2) threshold minimum and maximum for the red channel were set to 3 and 255 respectively, 3) filter for the hue was set at +9.
