# Supplementary figures and images for: The ROP vesicle release factor is required in adult Drosophila glia for normal circadian behavior
Source: Front Cell Neurosci. 2015 Jul 3;9:256. doi: 10.3389/fncel.2015.00256 (PMC4490253; doi:10.3389/fncel.2015.00256)

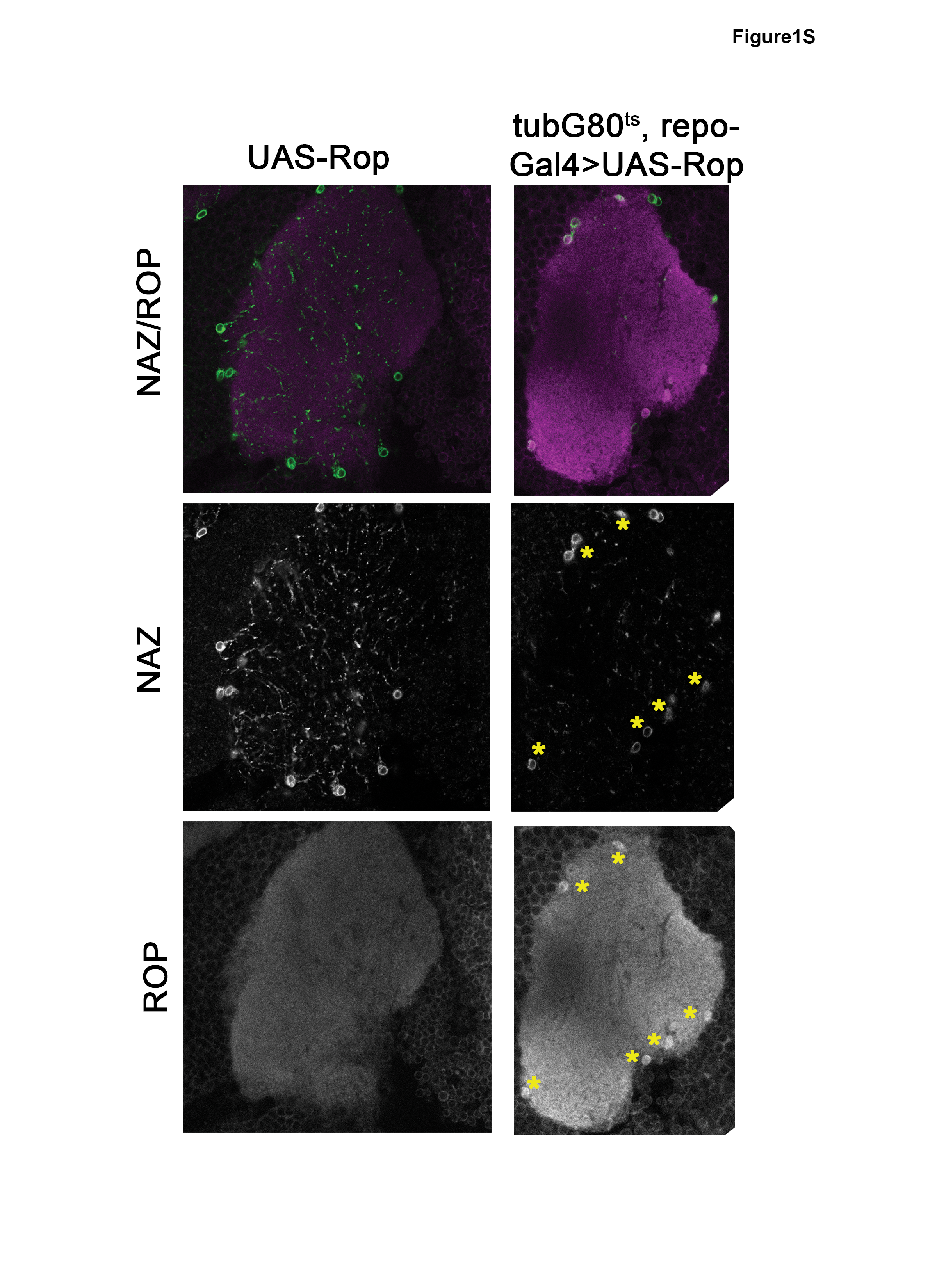

Supplement: Supplementary file 2 [file Image1.TIF]

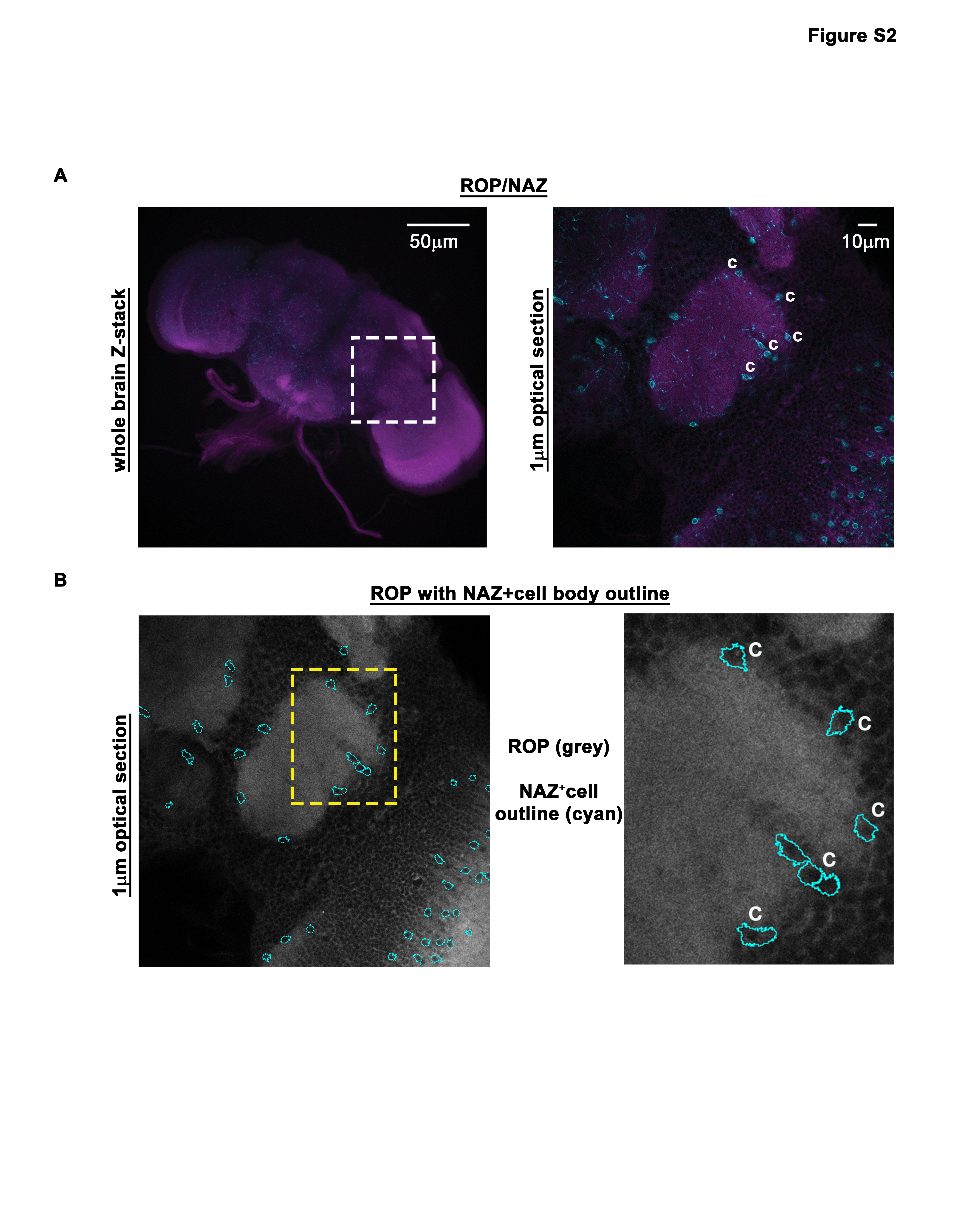

Supplement: Supplementary file 3 [file Image2.TIF]

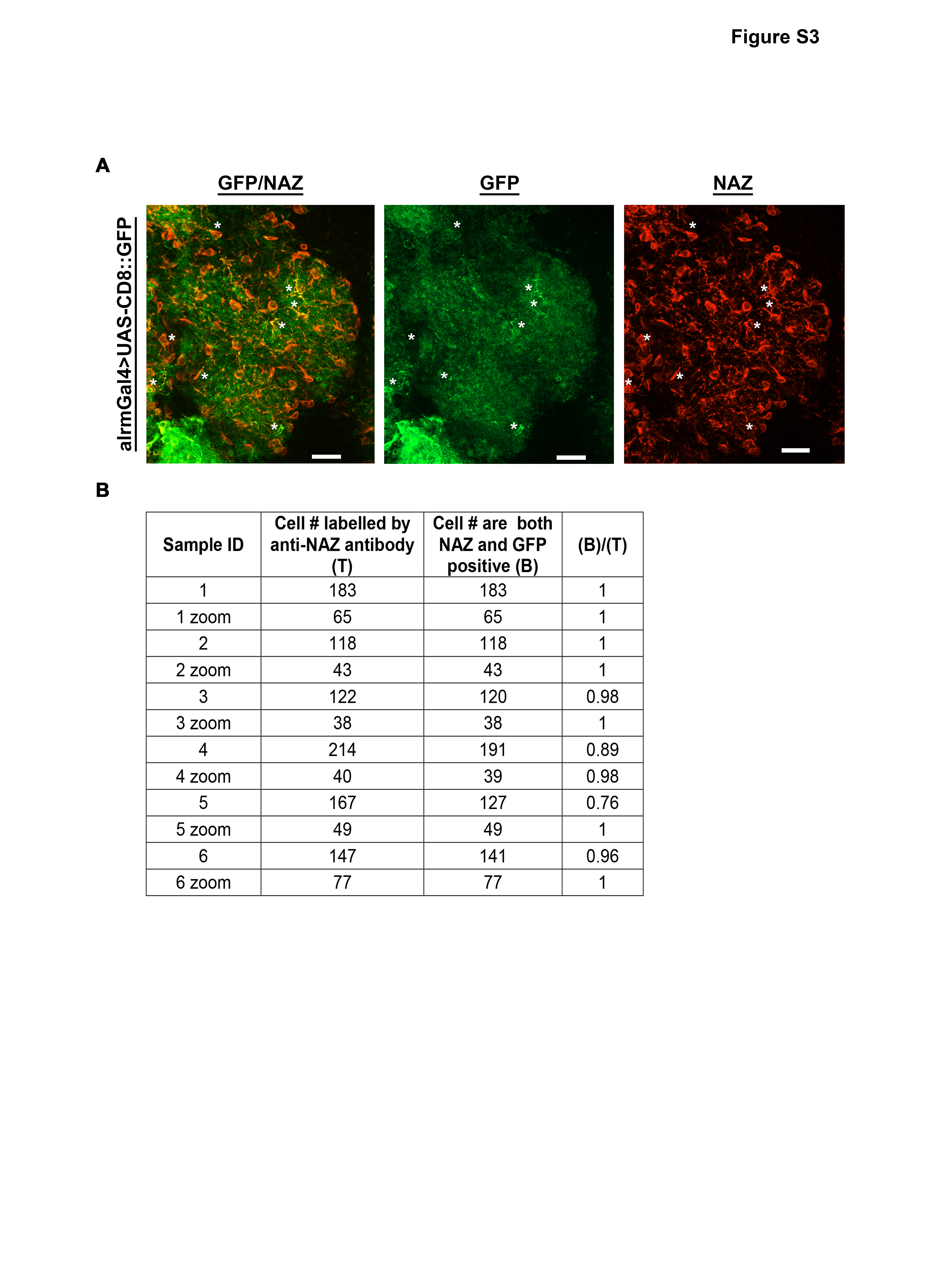

Supplement: Supplementary file 4 [file Image3.TIF]
